# Mortality among young adults in intensive care unit diagnosed with SARS-CoV-2 infection

**DOI:** 10.1590/0034-7167-2025-0141

**Published:** 2026-07-06

**Authors:** Marina Melo Martin, José Edilson de Oliveira, Priscila Gama Silva, Stella Sousa de Queiroz, Talita Andrade Santos, Dulce Aparecida Barbosa, Cassiane Dezoti Fonseca, Carla Roberta Monteiro Miura

**Affiliations:** I Universidade Federal de São Paulo. São Paulo, São Paulo, Brazil

**Keywords:** Young Adult, Critical Care, Intensive Care Units, Coronavirus Infections, COVID-19., Adulto Joven, Cuidados Críticos, Unidades de Cuidados Intensivos, Infecciones por Coronavirus, COVID-19.

## Abstract

**Objectives:**

to characterize young adults admitted to Intensive Care Units due to SARS-CoV-2 infection regarding demographic and clinical data and risk factors for mortality.

**Methods:**

quantitative, longitudinal, retrospective, and analytical research involving 105 adult patients, aged 20 to 40, in intensive care after confirmed SARS-CoV-2. It was conducted at a university hospital in São Paulo between April 2021 and December 2022.

**Results:**

the mortality rate was 27.6%. Sex, ethnicity, emergency room origin, hemodynamic instability, and vasoactive drug, mechanical ventilation, prophylactic heparin, and corticosteroids use were associated with mortality. Associated markers were C-reactive protein, lactate, and lymphocytes. Risk factors included increased lactate and days of vasoactive drug use.

**Conclusions:**

it was possible to outline the demographic and clinical profile of young adults admitted to the Intensive Care Unit due to COVID-19, and to know the variables predicting mortality in this population.

## RESUMEN

**Objetivos:**

caracterizar a los adultos jóvenes ingresados en Unidades de Cuidados Intensivos por infección por SARS-CoV-2 en cuanto a datos demográficos y clínicos, así como a los factores de riesgo de mortalidad.

**Métodos:**

estudio cuantitativo, longitudinal, retrospectivo y analítico con 105 pacientes adultos, de 20 a 40 años, ingresados en Unidades de Cuidados Intensivos tras infección confirmada por SARS-CoV-2. El estudio se realizó en hospital universitario de São Paulo entre abril/2021 y diciembre/2022.

**Resultados:**

la tasa de mortalidad fue del 27,6%. Sexo, etnia, procedencia de urgencias, inestabilidad hemodinámica, y uso de fármacos vasoactivos, ventilación mecánica, heparina profiláctica y corticosteroides se asociaron con la mortalidad. Los marcadores asociados fueron proteína C reactiva, lactato y linfocitos. Los factores de riesgo fueron aumento de los niveles de lactato y días de consumo de fármacos vasoactivos.

**Conclusiones:**

se logró establecer el perfil demográfico y clínico de los adultos jóvenes ingresados en la Unidad de Cuidados Intensivos por COVID-19, y comprender las variables predictoras de mortalidad en esta población.

## INTRODUCTION

The World Health Organization declared the coronavirus a pandemic on March 11, 2020. Since the first confirmed case in Brazil (February 26, 2020) to date (July 3, 2024), 6,862,680 cases have been confirmed in the state of São Paulo, with 38,828,259 confirmed cases in Brazil. The Southeast was the region of Brazil with the highest number of COVID-19 cases, with approximately 15,507,339 cases to date^(1)^. Brazil ranks fifth in the number of confirmed cases of COVID-19, behind only Germany (38,486,260), France (40,138,560), and India (44,998,565), with the United States having the highest number of confirmed cases (108,602,115)^(2)^.

Symptoms range from asymptomatic to critical cases, in which the main complications are acute respiratory distress syndrome, severe respiratory failure, severe pneumonia, secondary infection, sepsis, septic shock, acute respiratory distress syndrome, severe respiratory failure, hypoxia, renal failure, and multiple organ dysfunction, involving the need for respiratory support and admissions to Intensive Care Units (ICUs)^(3,4)^.

According to Pontes, patients with comorbidities, especially cardiovascular diseases, had a higher chance of death. Older adults, who constitute a vulnerable population, and patients with chronic conditions such as diabetes and cardiovascular or pulmonary diseases are not only at greater risk of developing serious illnesses but also of dying from them. In populations over 60, comorbidities are a risk factor. The physiological changes of aging and age-related comorbidities, such as heart and lung disease, diabetes, dementia, and polypharmacy, are associated with poor outcomes in older patients^(4)^.

However, this association with other comorbidities makes the younger population also susceptible to developing complications^(5)^. With the rejuvenation of the pandemic, an event that occurred in 2021, young and middle-aged adults began to represent a growing proportion of patients in ICUs, with the largest increase in the number of deaths from COVID-19 being in the youngest age group, from 20 to 29 years old^(6)^. Freitas (2021)^(7)^ attributed this phenomenon to several factors, including the emergence of variants with greater transmissibility and virulence, such as the Gamma variant (P.1), initially identified in Brazil, which also demonstrated a greater impact among younger individuals without significant comorbidities.

The first dose of the vaccine in Brazil was administered on January 17, 2021. Initially, the Ministry of Health chose to prioritize the vaccination of certain groups, such as healthcare professionals, older adults, and people with comorbidities. These data were reflected in the high ICU bed occupancy rates across all states, along with a reduction in the number of severe cases and ICU admissions. ^(8)^.

The Health Department points out that, in 2022, the risk of death from COVID-19 among young people aged 12 to 29 was 3.2 times higher for those who were not vaccinated with any dose, compared to those who had at least the complete primary schedule (two doses or a single dose)^(3)^.

The results of this study aim to expand knowledge about COVID-19. It is believed that by understanding the clinical profile of young adults in intensive care due to COVID-19 complications and the factors associated with prognosis, it will be possible to identify aspects that support planning for effective clinical management of the condition, aiming for better outcomes for this population.

## OBJECTIVES

To characterize young adults admitted to ICUs due to a diagnosis of SARS-CoV-2 infection regarding demographic and clinical data, verifying the variables that are associated and that behave as risk factors for mortality in this age group.

## METHODS

### Ethical aspects

The study was carried out in accordance with the provisions of Resolution 466/2012, which deals with research involving human beings, and the research project was approved by the Research Ethics Committee of the proposing institution, with no need to sign the Informed Consent Form, as it used secondary data.

### Study design, site and period

This is a quantitative, longitudinal, retrospective, and analytical study. The study design and description were conducted in accordance with guidelines established by STrengthening the Reporting of Observational studies in Epidemiology. The study setting included ICUs specialized in the care of COVID-19 patients at a university hospital in the state of São Paulo, a leading institution in teaching, research, and healthcare. Data collection ran from April 2021 to December 2022.

### Population, sample, inclusion and exclusion criteria

For convenience, we included young adult patients (aged 20 to 40 years) admitted to ICUs for at least 24 hours, with a primary diagnosis of SARS-CoV-2 infection confirmed by a reactive reverse transcriptase-polymerase chain reaction test. Patients whose electronic medical records were incomplete were excluded from the study. No sample size calculation was performed. All eligible patients were included in the study, which may limit the generalizability of the findings.

### Study protocol

Data were collected through electronic medical records after approval by the Research Ethics Committee, and the data were entered into the Research Electronic Data Capture data platform.

For this study, death was considered the dependent variable. Independent variables were classified as: demographic and health data (age, sex, race/ethnicity, and self-reported comorbidities); clinical data at admission (unit of origin, such as ward or emergency room; Glasgow Coma Scale score; respiration (spontaneous, with devices, or mechanical ventilation (MV)); hemodynamic instability (yes/no), defined as the need for vasopressors; laboratory data (C-reactive protein (CRP), D-dimer, lactate, lymphocytes, and blood gas analysis); clinical data of hospitalization (length of ICU stay (days), MV (no/yes - time in days), vasoactive drugs (VADs) (no/yes - time in days), pharmacological management (corticosteroid, immunoglobulin, hydroxychloroquine, oseltamivir, tocilizumab, prophylactic heparin and azithromycin), organ dysfunction (no/yes), defined by subjective clinical assessment, sepsis (no/yes), defined by clinical and laboratory criteria, delirium (no/yes), verified through the survey of daily records of the Confusion Assessment Method for the Intensive Care Unit^(9)^, and acute kidney injury (AKI) (no/yes), defined by the Kidney Disease Improving Global Outcomes (KDIGO) guidelines^(10)^; and outcome (ICU discharge/ICU death).

### Analysis of results and statistics

Data were grouped, transferred, and analyzed in R version 4.3.1. Quantitative variables were presented as central tendency and dispersion, and qualitative variables as absolute values and percentages. Numerical variables were analyzed using the Shapiro-Wilk normality test. The Mann-Whitney and Student’s t-tests were used to compare the median values of quantitative variables and outcome. Fisher’s exact and chi-square tests were used to compare the proportions of qualitative variables and death. A p-value of 5% was adopted as the significance level.

To assess death-related variables, the logistic model was used. Covariates were those that were observed to be related to death through statistical tests. The model was readjusted until all variables were found to be significant at the 5% level. The Odds Ratio presented was from the logistic model with variables significant at the 5% level.

## RESULTS

### Sample demographic and clinical characterization

This study consisted of 105 young adult patients admitted to the ICU with a diagnosis of COVID-19, the majority of whom were male (61%), white (54.3%) and had a mean age of 33 years (minimum of 21 and maximum of 40 years).

Among young adults admitted to the ICU due to COVID-19 infection, 62% had some comorbidity. Cardiovascular comorbidities accounted for 38% of all comorbidities, with hypertension (34.2%) standing out. Upon admission, 66.7% of individuals were referred from the emergency room, and only 25.71% were breathing spontaneously, with non-invasive ventilation being the most commonly used modality (27.62%).

During their ICU stay, almost half of the individuals (43.81%) presented some type of acid-base disturbance, with metabolic acidosis and respiratory alkalosis being the most common. Moreover, 64.76% presented hemodynamic instability, justified by the need for vasopressors. VADs were used in 64% of patients, with norepinephrine being the most prevalent (58%). Invasive MV was initiated in 64.7% of patients, with a median duration of use of five days. The most commonly used pharmacological management was heparin (81%), followed by corticosteroids (79%).

Concerning complications, sepsis was identified in 64.7% of patients, with a predominance of pulmonary focus (57%). AKI was present in 49% of cases, with KDIGO 3 being the most prevalent stratification (36.1%), and renal replacement therapy (RRT) was required in approximately 47% of patients. The incidence of delirium was 11%.

The mean length of stay in intensive care was 16 days, with a range from two to 104 days.

### Mortality in the Intensive Care Unit and associated factors

The mortality rate among young adults was 27.6%. As shown in Table 1, the demographic variables that showed an association with mortality were sex (p = 0.036), with a higher prevalence of death among females, and ethnicity (p = 0.057), with 58.3% of deaths among black people, as shown in Table 1.

**Table 1:** Distribution of young adults admitted to intensive care due to COVID-19 according to demographic characteristics, history, and their association with mortality, São Paulo, São Paulo, Brazil, 2024 (N=105)

Characteristics	General	ICU discharge	Death	*p* value
**Age (years)**				
Min.-Max.	21-40	21-40	24-40	0.832#
Median	34	34.5	34	
Mean (+-SD)	33	33.11	33.55	
**Sex**				
Female Male	41 64	25 (61%) 51 (80%)	16 (39%) 13 (20%)	0.036^*^
**Race**				
White Black	57 12	43 (75%) 5 (42%)	14 (25%) 7 (58%)	0.057^&^
Brown	36	28 (78%)	8 (22%)	
**Comorbidity**				
Cardiovascular Respiratory Metabolic Neurological	39 11 29 7	24 (62%) 6 (55%) 21 (72%) 6 (86%)	15 (38%) 5 (45%) 8 (28%) 1 (14%)	0.454^&^

#Mann-Whitney test;

* Chi-square test;

& Fisher’s exact test; ICU - Intensive Care Unit; SD - standard deviation.

Table 2 shows the clinical variables of hospitalization and their association with mortality. It is noted that the origin (p=0.045) is associated with mortality, in which a higher death rate (40%) was observed among patients coming from the ward. Hemodynamic instability (p=0.001), VAD use (p=0.001) and days of VAD use (0.001) also showed a significant association, in addition to the MV use (p=0.001) and days of MV use (p=0.001), with approximately 41.7% of deaths among patients who used MV.

**Table 2:** Distribution of young adults admitted to intensive care due to COVID-19 according to clinical characteristics and their association with mortality, São Paulo, São Paulo, Brazil, 2024 (N=105)

Characteristics	General	ICU discharge	Death	*p* value
**Origin** Infirmary Emergency room	35 70	21 (60%) 55 (79%)	14 (40%) 15 (21%)	0.045^*^
**Length of hospital stay (days)** Min.-Max. Median Mean (+-SD)	1-104 10 16	2-104 9.5 15.25	1-58 16 19.51	0.063^#^
**GCS on admission**				0.505^#^
Min.-Max. Median	10-15 15	10-15 15	14-15 15	
**Hemodynamic instability**				0.001^#^
Yes No	68 37	39 (57%) 37	29 (43%) 0	
**VAD use**				0.001^#^
Yes No	68 36	39 (57%) 36 (100%)	29 (43%) 0 (0%)	
**Days of VAD use**				0.001^#^
Min.-Max. Median Mean (+-SD)	0-58 2 6.67	0-43 1 4.4	1-58 8.5 13	
MV use^*^				0.001^*^
Yes No	68 37	40 (59%) 36 (97%)	28 (41%) 1 (3%)	
**Days of MV use**				0.001^#^
Min.-Max. Median Mean (+-SD)	0-63 5 9	0-63 3 7.2	1-58 12 15.1	
**Pharmacological management**				
Corticosteroid Immunoglobulin Oseltamivir	83 5 8	56 (67%) 3 (60%) 4 (50%)	27 (33%) 2 (40%) 4 (50%)	0.028^*^ 0.615^&^ 0.212^&^
Tocilizumab	1	0 (0%)	1 (100%)	0.276^&^
Heparin	85	67 (79%)	18 (21%)	0.002^*^
Azithromycin	34	25 (74%)	9 (26%)	0.855^*^
**Laboratory markers** **CRP**				0.031 ^†^
Min.-Max.	1.57-403.19	1.57-403.19	8.44-339.69	
Median	242.13	115.52	205.62	
**Lymphocytes**				0.046^#^
Min.-Max.	0-2641	4-2641	0-2535	
Median	939	1134	70	
**D-dimer**				0.475^#^
Min.-Max.	0.21-25	0.23-25	0.21-20	
Median	3.98	1.32	1.68	
**Lactate**				0.037^#^
Mín.-Máx.	5-238	5-38	6-238	
Median	14	12	15	

CRP - C-reactive protein; SD - standard deviation; ICU - Intensive Care Unit; GCS - Glasgow Coma Scale; VAD - vasoactive drug; MV - mechanical ventilation; #Mann-Whitney test;

* Chi-square test; &Fisher’s exact test; †Student’s t-test.

Among the pharmacological management options, prophylactic heparin and corticosteroids were used in approximately 80% of cases, with a significant association observed for death of p=0.002 and p=0.028, respectively.

Among laboratory markers, elevated CRP (p=0.031), lactate (p=0.037), and lymphocyte (p=0.046) values were associated with mortality, as shown in Table 2.

Complications associated with mortality were organ dysfunction (p=0.009), with 32.2% death and 100% discharge among those who did not present the dysfunction, and need for RRT (p=0.001) and sepsis (p=0.009), with approximately 44% and 41.71% death, respectively, as shown in Table 3.

**Table 3:** Distribution of young adults admitted to intensive care due to COVID-19 according to complications and their association with mortality, São Paulo, São Paulo, Brazil, 2024 (N=105)

Characteristics	General	ICU discharge	Death	*p* value
**Organic dysfunction**				
Yes	90	61 (68%)	29 (32%)	0.009^&^
No	15	15 (100%)	0 (0%)	
**Acid-base disorder**				
Yes	46	32 (70%)	14 (30%)	0.699^&^
No	54	40 (74%)	14 (26%)	
**AKI**				
KDIGO 1	6	3 (50%)	3 (50%)	0.572^&^
KDIGO 2	7	3 (43%)	4 (57%)	
KDIGO 3	38	24 (63%)	14 (37%)	
KDIGO 4	53	45 (85%)	8 (15%)	
**Need for renal replacement therapy**				
Yes	41	23 (56%)	18 (44%)	0.001^*^
No	47	41 (87%)	6 (13%)	
**Sepsis**				
Yes	68	40 (59%)	28 (41%)	0.009^*^
No	37	36 (97%)	1 (3%)	
***Delirium***				
Yes	11	8 (73%)	3 (27%)	1^&^
No	94	68 (72%)	26 (28%)	

ICU - Intensive Care Unit; AKI - acute kidney injury; KDIGO - Kidney Disease Improving Global Outcomes;

# Mann-Whitney test;

* Chi-square test; &Fisher’s exact test.

### Risk factors and prediction for mortality

Among the variables studied, increased lactate levels and days of VAD use were shown to be risk factors for death in the ICU, as shown in Table 4.

**Table 4:** Risk factors for COVID-19 mortality in young adults in intensive care, São Paulo, São Paulo, Brazil, 2024 (N=105)

	Odds Ratio	LL	UL	*p* value
**Intercept**	0.064	0.017	0.188	<0.001
**Lactate**	1.061	1.011	1.131	0.047
**VAD days**	1.120	1.058	1.198	<0.001

*VAD - vasoactive drug; LL - lower limit; UL - upper limit. Note: Odds Ratio only for variables with statistical significance of p value <0.05.*

It can be seen that for each one-unit increase in lactate, the chance of death increases by 6.1%. For each one-unit increase in VAD days, the chance of death increases by 12%. The model is adequate using the Hosmer-Lemeshow test (p-value = 0.378). The area under the ROC curve is 0.815, indicating excellent individual discrimination.

## DISCUSSION

In the cohort, a mortality rate of 27.6% was observed, higher than the value observed in the literature. According to Fiocruz, in 2021, COVID-19 mortality among adults aged 20 to 39 ranged from 3.1% to 10.2%^(11)^. A study carried out in Manaus, Rio de Janeiro and São Paulo shows that, in 2020, the mortality rate varied from 14.64% to 18.28% in this same age group^(12)^. The high mortality in the present sample is probably due to death in the ICU, while the other studies considered general death (inor out-of-hospital), disregarding the severity of COVID-19 infection.

In the present study, sex and ethnicity were associated with death. The literature highlights higher mortality among individuals with non-white skin color, males, and those with comorbidities^(13)^.

Contrary to the results obtained, a meta-analysis found that, although men and women are at equal risk of infection, men are more susceptible to COVID-19 cases and mortality. This justification would be due to the fact that women have the advantage of having a higher number of CD4 T cells and B lymphocytes^(14)^. However, this benefit may be mitigated under certain clinical circumstances, such as pregnancy, immunosuppressive medication use, or the presence of poorly controlled comorbidities. Furthermore, social determinants of health and underreporting of symptoms in women may also contribute to worse outcomes, even in groups that theoretically would be at lower risk. Another important point to consider is the possible influence of sampling bias or unadjusted confounding factors, such as differences in the severity of clinical symptoms upon ICU admission, comorbidity profile, and specific obstetric conditions.

The higher mortality rates among black people from COVID-19 observed in this study is consistent with widely documented evidence in the scientific literature. A study that observed mortality from COVID-19 also highlighted that black individuals in Brazil and in all regions except the North had a higher risk of hospital mortality^(15)^. This disparity cannot be attributed solely to biological factors, but rather to structural inequalities that disproportionately affect black populations^(16)^. African American/black and Hispanic populations face a disproportionate burden of SARS-CoV-2 infections and COVID-19-related mortality, but not higher than the case fatality rates. A literature review indicates that lack of access to healthcare and exposure factors in this population underlie the observed disparities more than susceptibility due to comorbid conditions^(17)^.

Cardiovascular comorbidities accounted for 38% of all comorbidities among the study subjects, with hypertension standing out (34.2%). Metabolic comorbidities accounted for 27% of all comorbidities, with diabetes mellitus being the most common (35%). In a study by Zhou *et al*. (2020), comorbidities were present in almost half of patients, with hypertension being the most common comorbidity, followed by diabetes and coronary heart disease. The likelihood of in-hospital death was higher in patients with diabetes or coronary heart disease^(18)^.

In the present study, no significant association was observed between death and comorbidities. One possible explanation for this finding may be related to the sample size or the heterogeneity of comorbidities. Furthermore, it is possible that factors such as individual inflammatory response, length of hospital stay, and timely access to intensive care play a more decisive role in the mortality of young adults, reducing the relative weight of comorbidities alone. Selection bias should also be considered, since all individuals in the sample were already in critical condition at the length of ICU admission, which may have homogenized the mortality risk, regardless of prior clinical history.

A sepsis rate of 64.7% was observed, with pulmonary bacterial coinfection being the most frequent (57%). A similar rate (60%) was observed in a study conducted in New York, which involved 152 patients with COVID-19^(19)^. Another study involving COVID-19 patients with a mean age of 47 years also showed pulmonary sepsis as the main complication among patients^(16)^. Ventilator-associated pneumonia (VAP) may contribute to this rate of lung infection. A study involving mechanically ventilated COVID-19 patients showed a VAP rate of 54%^(20)^.

MV use was a variable related to mortality (p=0.001), as confirmed by other studies, in which the mortality rate among those mechanically ventilated ranged from 81% to 97%^(21-23)^.

A study conducted in Wuhan, which analyzed the relationship between COVID-19 and MV at the beginning of the emergence of cases, highlighted MV as the main supportive treatment for critically ill patients. Approximately 71% required MV, and 67% had acute respiratory distress syndrome^(18)^.

High PEEP, prolonged respiratory support use caused by impaired gas exchange, and severe hypoxemia in COVID-19 patients are associated with the onset of AKI and increased morbidity and mortality. Another possible explanation for AKI rates is directly related to VAD use, which cause severe vasoconstriction, decreasing renal flow^(24)^.

Young adults with COVID-19 who developed AKI, especially in the KDIGO 3 stratification, had a high number of deaths, as shown in the literature^(20)^. A study observed a very similar mortality rate among those who developed AKI (44.63%)^(25)^.

The mortality rate among those who used RRT was 44%, and the literature indicates rates ranging from 36% to 90%^(26)^.

The first update of the Surviving Sepsis campaign guidelines on managing adults with COVID-19 in the ICU strongly recommends using a higher positive end-expiratory pressure (PEEP) strategy^(27)^.

About 65% of young adults used at least one VAD. Studies show that VAD use among COVID-19 patients treated in the ICU ranges from 28% to 94%^(28,29)^, norepinephrine being the first-line vasopressor, and vasopressin, the second-line agent, as evidenced in the clinical management of the sample studied^(30,31)^.

The finding of a statistically significant association between prophylactic heparin (p=0.002) and corticosteroid use (p=0.028), and the death outcome deserves careful analysis. Although both treatments are included in COVID-19 treatment guidelines with the aim of reducing thromboembolic and inflammatory complications, the association with higher mortality may reflect severity bias, meaning that more severely ill patients are more likely to receive these interventions. A combination of a dysregulated immune response, with immunosuppression induced by viruses and drugs, such as corticosteroids, can contribute to the emergence of secondary infections in patients with COVID-19, which can culminate in higher mortality rates^(32)^.

Among laboratory markers, elevated CRP (p=0.031), lactate (p=0.037), and lymphocyte (p=0.046) levels were associated with mortality. In a study by Zhou *et al*., lactate levels were similarly elevated in non-survivors compared with survivors throughout the clinical course, as were D-dimer levels, unlike the findings of the present study^(18)^.

Elevated serum lactic acid levels are commonly associated with a poor prognosis and are used as a sensitive index of shock. Many conditions can increase lactic acid levels, including anaerobic metabolism, glycolysis, cytokine storm, and liver dysfunction. Therefore, an important way to detect sepsis in COVID-19 is through biomarkers such as lactic acid^(22)^. In addition, when observing the rate of VAD use obtained, it can be inferred that many of the patients in the sample studied developed septic shock.

In a study conducted in the United States that assessed the occurrence of sepsis and hyperlactatemia in patients with COVID-19 admitted to intensive care, it was observed that increased lactate is not a good prognosis in COVID-19, with mortality rates being higher than those of other types of sepsis^(33)^.

In this study, among the variables studied, increased lactate levels and days of VAD use were shown to be risk factors for death in the ICU.

Studies have pointed to other risk factors not highlighted in this study, such as males, hypertension, diabetes, chronic obstructive pulmonary disease, cardiovascular or oncological diseases, high CRP levels, and D-dimer. It should be noted that these studies did not assess the adult population with age stratification^(34,35)^.

Other factors can be decisive in increasing the risk of death regardless of age. The lack of adequate public prevention policies and the low responsiveness of the healthcare network demonstrate a context of significant socioeconomic inequality and unequal access to healthcare services, factors that directly influence COVID-19 mortality in Brazil^(12)^.

### Study limitations

Study limitations include data loss due to missing records in electronic medical records. Furthermore, the results may not represent reality, as the study was conducted at a single center. Furthermore, it was difficult to adequately compare findings due to the scarcity of studies with results stratified by age group, specifically involving young adults or patients in the exclusive ICU setting, reinforcing the knowledge gap. The number of outcomes (n=29 deaths) imposes restrictions on the number of variables adjusted simultaneously, which may impact the robustness of the model.

### Contributions to nursing, health or public policy

This study allowed us to outline the demographic and clinical profile of young adults admitted to the ICU due to COVID-19, as well as to understand associated variables and predictors of mortality in this population. The analysis of data from this study is relevant, as it adds to existing knowledge about COVID-19, helping to guide management and care measures, and contributing to multidisciplinary clinical practice, aiming for better outcomes.

## CONCLUSIONS

A mortality rate of 27.6% was obtained among young adults. Variables associated with death were sex (p=0.036), ethnicity (p=0.0057), origin (p=0.045), hemodynamic instability (p=0.001), VAD use (p=0.001), MV use (p=0.001), prophylactic heparin use (p=0.002) and corticosteroid use(p=0.028), in addition to increased values of CRP (p=0.031), lactate (p=0.037), and lymphocytes (p=0.046).

Complications associated with mortality were presenting organ dysfunction (p=0.009), sepsis (p=0.009) and requiring RRT (p=0.001).

Increased lactate levels and days of VAD use were shown to be risk factors for death in the ICU.

## Data Availability

The research data are available within the article.
