# Supplementary material for: Mortality among young adults in intensive care unit diagnosed with SARS-CoV-2 infection
Source: Rev Bras Enferm. 2026 Jul 6;79:e20250141. doi: 10.1590/0034-7167-2025-0141 (PMC13336224; doi:10.1590/0034-7167-2025-0141)
Supplement: Translation

# Translation: Mortalidade entre adultos jovens em unidade de terapia intensiva com diagnóstico de infecção por SARS-CoV-2

**DOI:** 10.1590/0034-7167-2025-0141pt

**Authors:** Marina Melo Martin, José Edilson de Oliveira, Priscila Gama Silva, Stella Sousa de Queiroz, Talita Andrade Santos, Dulce Aparecida Barbosa, Cassiane Dezoti Fonseca, Carla Roberta Monteiro Miura

**Affiliations:** I Universidade Federal de São Paulo. São Paulo, São Paulo, Brasil

**Keywords:** Adulto Jovem, Cuidados Críticos, Unidade de Terapia Intensiva, Infecções por Coronavírus, COVID-19.

## Abstract

**Objetivos:**

caracterizar adultos jovens admitidos nas Unidades de Terapia Intensiva por infecção por SARS-CoV-2 quanto aos dados demográficos, clínicos e fatores de risco para mortalidade.

**Métodos:**

pesquisa quantitativa, longitudinal, retrospectiva e analítica, com 105 pacientes adultos, de 20 a 40 anos, em terapia intensiva mediante confirmação por SARS-CoV-2. Foi realizado em hospital universitário, em São Paulo, entre abril/2021 e dezembro/2022.

**Resultados:**

a taxa de mortalidade foi de 27,6%. Sexo, etnia, procedência do pronto-socorro, instabilidade hemodinâmica, e uso de droga vasoativa, de ventilação mecânica, de heparina profilática e de corticoide estiveram associados à mortalidade. Os marcadores associados foram a proteína C reativa, lactato e linfócitos. Os fatores de risco foram o aumento do lactato e dias de uso de droga vasoativa.

**Conclusões:**

foi possível traçar o perfil demográfico e clínico de adultos jovens internados em Unidade de Terapia Intensiva por COVID-19, e conhecer as variáveis preditoras de mortalidade nessa população.

## INTRODUÇÃO

A Organização Mundial da Saúde declarou pandemia por coronavírus no dia 11 de março de 2020. Desde o primeiro caso confirmado no Brasil (26 de fevereiro de 2020) até o momento (03 de julho de 2024), 6.862.680 casos foram confirmados no estado de São Paulo, com 38.828.259 casos confirmados no Brasil. Sudeste foi a região do Brasil que apresentou maior número de casos de COVID-19, com cerca de 15.507.339 casos até o momento^(1)^. O Brasil se encontra em quinto lugar no número de casos confirmados de COVID-19, ficando atrás somente da Alemanha (38.486.260), França (40.138.560) e Índia (44.998.565), sendo os Estados Unidos com o maior número de casos confirmados (108.602.115)^(2)^.

Os sintomas variam desde o caso assintomático até o caso crítico, em que as principais complicações são síndrome do desconforto respiratório agudo, insuficiência respiratória grave, pneumonia grave, infecção secundária, sepse, choque séptico, síndrome do desconforto respiratório agudo, insuficiência respiratória grave, hipóxia, insuficiência renal, e disfunção de múltiplos órgãos, envolvendo a necessidade de suporte respiratório e internações em Unidades de Terapia Intensiva (UTIs)^(3,4)^.

Segundo Pontes, os pacientes com comorbidades, especialmente cardiovasculares, tiveram maior chance de óbito. Os idosos, que constituem população vulnerável, e pacientes com condições crônicas, como diabetes e doenças cardiovasculares ou pulmonares, não somente correm maior risco de desenvolver doenças graves, mas também de ir a óbito pela doença. Nas populações acima de 60 anos, as comorbidades se apresentam como fator de risco. As mudanças fisiológicas do envelhecimento e das comorbidades relacionadas à idade, como doenças cardíacas e pulmonares, diabetes, demência e polifarmácia, estão associadas a resultados desfavoráveis em pacientes mais idosos^(4)^.

No entanto, essa associação com outras comorbidades torna a população mais jovem também suscetível a desenvolver complicações^(5)^. Com o rejuvenescimento da pandemia, evento que ocorreu em 2021, adultos jovens e de meia idade passaram a representar uma proporção crescente de pacientes nas UTIs, em que o maior aumento no número de mortes por COVID-19 esteve na faixa etária, dos mais jovens, de 20 a 29 anos^(6)^. Freitas (2021)^(7)^ atribuiu esse fenômeno a múltiplos fatores, incluindo o surgimento de variantes com maior transmissibilidade e virulência, como a variante Gama (P.1), inicialmente identificada no Brasil, que demonstrou maior impacto também entre indivíduos mais jovens e sem comorbidades significativas.

A primeira dose da vacina no Brasil foi aplicada em 17 de janeiro de 2021. Inicialmente, o Ministério da Saúde optou por priorizar a vacinação de determinados grupos, como profissionais de saúde, idosos e pessoas com comorbidades. Esses dados refletiram nas altas taxas de ocupação dos leitos de UTI em todos os estados, com a redução do número de casos graves e internações em UTI^(8)^.

A Secretaria da Saúde aponta que, em 2022, o risco de morte por COVID-19 entre os jovens de 12 a 29 anos foi 3,2 vezes maior para quem não foi vacinado com nenhuma dose, em comparação com quem tinha, ao menos, o esquema primário completo (duas doses ou dose única)^(3)^.

Busca-se, com os resultados deste estudo, ampliar o conhecimento sobre a COVID-19. Acredita-se que, na medida em que se reconhecem o perfil clínico do adulto jovem em terapia intensiva decorrente de complicações da COVID-19 e os fatores associados ao prognóstico, é possível levantar aspectos que subsidiem o planejamento de um bom manejo clínico da condição com vistas a melhores desfechos para essa população.

## OBJETIVOS

Caracterizar adultos jovens admitidos nas UTIs por diagnóstico de infecção por SARS-CoV-2 quanto aos dados demográficos e clínicos, verificando as variáveis que se mostram associadas e que se comportam como fatores de risco para mortalidade nesse grupo etário.

## MÉTODOS

### Aspectos éticos

O estudo ocorreu em conformidade com o preconizado pela Resolução n^o^ 466/2012, que versa sobre pesquisa em seres humanos, sendo o projeto de pesquisa aprovado pelo Comitê de Ética em Pesquisa da instituição proponente, havendo dispensa da assinatura do Termo de Consentimento Livre e Esclarecido, por utilizar dados secundários.

### Desenho, local e período do estudo

Trata-se de pesquisa quantitativa, com delineamento longitudinal, retrospectivo e analítico, cuja elaboração e descrição foram realizadas conforme os pressupostos preconizados pelo *STrengthening the Reporting of OBservational studies in Epidemiology*. O cenário do estudo incluiu as UTIs especializadas no atendimento a pacientes com COVID-19 de um hospital universitário no estado de São Paulo, referência em ensino, pesquisa e assistência. O período de coleta de dados ocorreu de abril de 2021 a dezembro de 2022.

### População, amostra, critérios de inclusão e exclusão

Por conveniência, foram incluídos pacientes adultos jovens (idade entre 20 e 40 anos) internados nas UTIs por, no mínimo, 24 horas, com diagnóstico primário de infecção por SARS-CoV-2 confirmado mediante o resultado reagente ao teste de reação de transcriptase reversa seguida de reação em cadeia da polimerase. Foram excluídos do estudo pacientes cujos prontuários eletrônicos se encontravam incompletos. Não foi realizado cálculo amostral. Todos os pacientes elegíveis foram incluídos no estudo, o que pode gerar uma limitação na generalização dos achados.

### Protocolo do estudo

Os dados foram levantados por meio de prontuário médico eletrônico após a aprovação do Comitê de Ética em Pesquisa, e os dados foram inseridos na plataforma de dados *Research Electronic Data Capture*.

Para o presente estudo, foi considerada, como variável dependente, a ocorrência de óbito. As variáveis independentes foram classificadas em: dados demográficos e de saúde (idade, sexo, raça/cor e comorbidades autodeclaradas); dados clínicos da admissão (unidade de procedência, como enfermaria ou pronto-socorro, Escala de Coma de Glasgow, respiração (espontânea, com dispositivos ou ventilação mecânica (VM)), instabilidade hemodinâmica (sim/não), definida pela necessidade de vasopressores); dados laboratoriais (proteína C reativa (PCR), D-dímero, lactato, linfócitos e gasometria); dados clínicos da internação (tempo de internação na UTI (dias), VM (não/sim - tempo em dias), drogas vasoativas (DVAs) (não/sim - tempo em dias), manejo farmacológico (corticoide, imunoglobulina, hidroxicloroquina, oseltamivir, tocilizumabe, heparina profilática e azitromicina), disfunção orgânica (não/sim), definida por avaliação clínica subjetiva, sepse (não/sim), definida por critérios clínicos e laboratoriais, *delirium* (não/sim), verificado através do levantamento dos registros diários do *Confusion Assessment Method for the Intensive Care Unit* ^(9)^, e lesão renal aguda (LRA) (não/sim), definida pelas diretrizes *Kidney Disease Improving Global Outcomes* (KDIGO)^(10)^; e desfecho (alta da UTI/óbito na UTI).

### Análise dos resultados e estatística

Os dados foram agrupados, transferidos e analisados no R, versão 4.3.1. As variáveis quantitativas foram apresentadas em tendência central e dispersão, e as variáveis qualitativas, em valores absolutos e percentuais. As variáveis numéricas foram analisadas pelo teste de normalidade de Shapiro-Wilk. Para comparação dos valores medianos das variáveis quantitativas e desfecho, foram utilizados os testes de Mann-Whitney e t de Student. Para comparação das proporções das variáveis qualitativas e óbito, foram utilizados os testes exato de Fisher e qui-quadrado. Adotou-se como nível de significância um valor de p de 5%.

Para verificar as variáveis relacionadas ao óbito, usou-se o modelo logístico. Foram utilizadas como covariáveis aquelas em que foi observada relação com a morte por meio de testes estatísticos. Reajustou-se o modelo até encontrar aquele com todas as variáveis significativas a 5%. A razão de chances apresentada foi do modelo logístico com variáveis significativas a 5%.

## RESULTADOS

### Caracterização demográfica e clínica da amostra

Este estudo foi composto de 105 pacientes adultos jovens internados na UTI com diagnóstico de COVID-19, em que a maioria era do sexo masculino (61%), de etnia branca (54,3%) e com idade média de 33 anos (mínima de 21 e máxima de 40 anos).

Entre os adultos jovens admitidos na UTI por infecção por COVID-19, 62% possuíam alguma comorbidade. As comorbidades cardiovasculares representaram 38% do total das comorbidades, com destaque para a hipertensão arterial sistêmica (34,2%). Na admissão, 66,7% dos indivíduos eram provenientes do pronto-socorro, e apenas 25,71% apresentavam respiração espontânea, sendo a ventilação não invasiva a modalidade mais empregada (27,62%).

No decorrer da internação na UTI, quase metade dos indivíduos apresentou algum tipo de distúrbio ácido-básico (43,81%), sendo acidose metabólica e alcalose respiratória os mais frequentes, e 64,76% apresentaram instabilidade hemodinâmica, justificada pela necessidade de vasopressores. DVAs foram utilizadas em 64% dos pacientes, sendo mais prevalente o uso de noradrenalina (58%). A VM invasiva foi iniciada em 64,7% dos pacientes, sendo o tempo mediano de uso de cinco dias. O manejo farmacológico mais utilizado foi o emprego da heparina (81%), seguido de corticoide (79%).

Quanto às complicações, a sepse foi identificada em 64,7% dos pacientes, com predominância do foco pulmonar (57%). A LRA esteve presente em 49% dos casos, sendo a estratificação mais prevalente a KDIGO 3 (36,1%), e o uso de terapia renal substitutiva (TRS) foi necessário por aproximadamente 47% dos internados. Observou-se uma incidência de *delirium* de 11%.

O tempo médio de internação na terapia intensiva foi de 16 dias, com variação de dois a 104 dias.

### Mortalidade na Unidade de Terapia Intensiva e fatores associados

A taxa de mortalidade entre os adultos jovens foi de 27,6%. Conforme mostra a Tabela 1, as variáveis demográficas que mostraram associação com a mortalidade foram sexo (p =0,036), com maior prevalência de óbito no sexo feminino, e etnia (p =0,057), com 58,3% de óbito entre os negros, conforme mostra a Tabela 1.

**Tabela 1:** Distribuição dos adultos jovens admitidos em terapia intensiva por COVID-19 quanto às características demográficas, antecedentes e sua associação com mortalidade, São Paulo, São Paulo, Brasil, 2024 (N=105)

Características	Geral	Alta da UTI	Óbito	Valor de *p*
**Idade (anos)**				
Mín.-Máx.	21-40	21-40	24-40	0,832#
Mediana	34	34,5	34	
Média (+-DP)	33	33,11	33,55	
**Sexo**				
Feminino Masculino	41 64	25 (61%) 51 (80%)	16 (39%) 13 (20%)	0,036^*^
**Raça**				
Branca Negra	57 12	43 (75%) 5 (42%)	14 (25%) 7 (58%)	0,057^&^
Parda	36	28 (78%)	8 (22%)	
**Comorbidade**				
Cardiovascular Respiratória Metabólica Neurológica	39 11 29 7	24 (62%) 6 (55%) 21 (72%) 6 (86%)	15 (38%) 5 (45%) 8 (28%) 1 (14%)	0,454^&^

# Teste de Mann-Whitney; ^^*^^Teste qui-quadrado;

& Teste exato de Fisher; UTI - Unidade de Terapia Intensiva; DP - desvio padrão.

As variáveis clínicas da internação e sua associação com a mortalidade estão expressas na Tabela 2. Nota-se que a procedência (p=0,045) está associada à mortalidade, em que se observou maior taxa de óbito (40%) entre os pacientes provenientes da enfermaria. Instabilidade hemodinâmica (p=0,001), uso de DVA (p=0,001) e dias de uso de DVA (0,001) também apresentaram associação significativa, além do uso de VM (p=0,001) e dias de uso de VM (P=0,001), com cerca de 41,7% de óbito dos pacientes que fizeram uso de VM.

**Tabela 2:** Distribuição dos adultos jovens admitidos em terapia intensiva por COVID-19 quanto às características clínicas e sua associação com mortalidade, São Paulo, São Paulo, Brasil, 2024 (N=105)

Características	Geral	Alta da UTI	Óbito	Valor de *p*
**Procedência** Enfermaria Pronto-socorro	35 70	21 (60%) 55 (79%)	14 (40%) 15 (21%)	0,045^*^
**Tempo de internação (dias)** Mín.-Máx. Mediana Média (+-DP)	1-104 10 16	2-104 9,5 15,25	1-58 16 19,51	0,063^#^
**ECG na admissão**				0,505^#^
Mín.-Máx. Mediana	10-15 15	10-15 15	14-15 15	
**Instabilidade hemodinâmica**				0,001^#^
Sim Não	68 37	39 (57%) 37	29 (43%) 0	
**Uso de DVA**				0,001^#^
Sim Não	68 36	39 (57%) 36 (100%)	29 (43%) 0 (0%)	
**Dias de uso de DVA**				0,001^#^
Mín.-Máx. Mediana Média (+-DP)	0-58 2 6,67	0-43 1 4,4	1-58 8,5 13	
Uso de VM^*^				0,001^*^
Sim Não	68 37	40 (59%) 36 (97%)	28 (41%) 1 (3%)	
**Dias de uso de VM**				0,001^#^
Mín.-Máx. Mediana Média (+-DP)	0-63 5 9	0-63 3 7,2	1-58 12 15,1	
**Manejo farmacológico**				
Corticoide Imunoglobulina Oseltamivir	83 5 8	56 (67%) 3 (60%) 4 (50%)	27 (33%) 2 (40%) 4 (50%)	0,028^*^ 0,615^&^ 0,212^&^
Tocilizumabe	1	0 (0%)	1 (100%)	0,276^&^
Heparina	85	67 (79%)	18 (21%)	0,002^*^
Azitromicina	34	25 (74%)	9 (26%)	0,855^*^
**Marcadores laboratoriais** **PCR**				0,031 ^†^
Mín.-Máx.	1,57-403,19	1,57-403,19	8,44-339,69	
Mediana	242,13	115,52	205,62	
**Linfócitos**				0,046^#^
Mín.-Máx.	0-2641	4-2641	0-2535	
Mediana	939	1134	70	
**D-dímero**				0,475^#^
Mín.-Máx.	0,21-25	0,23-25	0,21-20	
Mediana	3,98	1,32	1,68	
**Lactato**				0,037^#^
Mín.-Máx.	5-238	5-38	6-238	
Mediana	14	12	15	

PCR - proteína C reativa; DP - desvio padrão; UTI - Unidade de Terapia Intensiva; ECG - Escala de Coma de Glasgow; DVA - droga vasoativa; VM - ventilação mecânica; #Teste de Mann-Whitney;

* Teste qui-quadrado;

& Teste exato de Fisher;

† Teste t de Student.

Entre as opções de manejo farmacológico, a heparina profilática e o corticoide foram empregados em cerca de 80% dos casos, sendo que se observou uma associação significativa para o óbito de p=0,002 e p=0,028, respectivamente.

Entre os marcadores laboratoriais, a elevação dos valores de PCR(p=0,031), lactato (p=0,037) e linfócitos (p=0,046) esteve associado à mortalidade, conforme mostra a Tabela 2.

As complicações associadas à mortalidade foram disfunção orgânica (p=0,009), com 32,2% de óbito e 100% de alta entre aqueles que não apresentaram a disfunção, e necessidade de TSR (p=0,001) e sepse (p=0,009), com cerca de 44% e 41,71% de óbito, respectivamente, conforme mostra a Tabela 3.

**Tabela 3:** Distribuição dos adultos jovens admitidos em terapia intensiva por COVID-19 quanto às complicações e sua associação com mortalidade, São Paulo, São Paulo, Brasil, 2024 (N=105)

Características	Geral	Alta da UTI	Óbito	Valor de *p*
**Disfunção orgânica**				
Sim	90	61 (68%)	29 (32%)	0,009^&^
Não	15	15 (100%)	0 (0%)	
**Distúrbio ácido-básico**				
Sim	46	32 (70%)	14 (30%)	0,699^&^
Não	54	40 (74%)	14 (26%)	
**LRA**				
KDIGO 1	6	3 (50%)	3 (50%)	0,572^&^
KDIGO 2	7	3 (43%)	4 (57%)	
KDIGO 3	38	24 (63%)	14 (37%)	
KDIGO 4	53	45 (85%)	8 (15%)	
**Necessidade de terapia de substituição renal**				
Sim	41	23 (56%)	18 (44%)	0,001^*^
Não	47	41 (87%)	6 (13%)	
**Sepse**				
Sim	68	40 (59%)	28 (41%)	0,009^*^
Não	37	36 (97%)	1 (3%)	
***Delirium***				
Sim	11	8 (73%)	3 (27%)	1^&^
Não	94	68 (72%)	26 (28%)	

UTI - Unidade de Terapia Intensiva; LRA - lesão renal aguda; KDIGO - Kidney Disease Improving Global Outcomes;

# Teste de Mann-Whitney;

* Teste qui-quadrado; &Teste exato de Fisher.

### Fatores de risco e predição para mortalidade

Entre as variáveis estudadas, mostraram-se como fatores de risco para óbito na UTI o aumento dos níveis de lactato e os dias de uso de DVA, conforme mostra a Tabela 4.

**Tabela 4:** Fatores de risco para mortalidade por COVID-19 em adultos jovens na terapia intensiva, São Paulo, São Paulo, Brasil, 2024 (N=105)

	Razão de chances	LI	LS	Valor de *p*
**Intercepto**	0,064	0,017	0,188	<0,001
**Lactato**	1,061	1,011	1,131	0,047
**Dias de DVA**	1,120	1,058	1,198	<0,001

*DVA - droga vasoativa; LI - limite inferior; LS - limite superior. Nota: razão de chances apenas das variáveis com significância estatística de valor de p <0,05.*

Nota-se que, a cada aumento de uma unidade em lactato, aumenta-se em 6,1% a chance de ir a óbito. A cada aumento de 1 unidade em dias de DVA, aumenta-se em 12% a chance de ir a óbito. O modelo é adequado pelo teste de Hosmer-Lemeshow (valor de p=0,378). A área abaixo da curva ROC é 0,815, indicando ótima discriminação dos indivíduos.

## DISCUSSÃO

Na coorte, foi observada taxa de mortalidade de 27,6%, maior que o valor observado na literatura. Segundo a Fiocruz, em 2021, a mortalidade por COVID-19 entre adultos de 20 a 39 anos variou de 3,1% a 10,2%^(11)^. Estudo realizado em Manaus, Rio de Janeiro e São Paulo mostra que, em 2020, a taxa de mortalidade variou de 14,64% a 18,28% nessa mesma faixa etária^(12)^. A alta mortalidade na presente amostra é provavelmente devido ao óbito em UTI, enquanto os demais estudos consideraram o óbito geral (intra ou extra-hospitalar), desconsiderando a gravidade da infecção por COVID-19.

No presente estudo, sexo e etnia apresentaram associação com o óbito. A literatura destaca uma maior mortalidade entre indivíduos com cor de pele não branca, sexo masculino e portador de comorbidades^(13)^.

Contrariamente aos resultados obtidos, metanálise apontou que, embora homens e mulheres corram risco equivalente de infecção, o sexo masculino é mais suscetível aos casos e mortalidade por COVID-19. Tal justificativa se daria pelo fato de o sexo feminino apresentar como vantagens maior quantidade de células TCD4 e linfócitos B^(14)^. No entanto, esse benefício pode ser atenuado em determinadas circunstâncias clínicas, como gravidez, uso de medicamentos imunossupressores ou presença de comorbidades mal controladas. Além disso, determinantes sociais de saúde e subvalorização de sintomas em mulheres também podem contribuir para piores desfechos, mesmo em grupos que teoricamente teriam menor risco. Outro ponto importante a considerar é a possível influência de viés amostral ou fatores confundidores não ajustados, como diferenças na gravidade do quadro clínico ao ingresso na UTI, perfil de comorbidades e condições obstétricas específicas.

A maior mortalidade de pessoas negras por COVID-19 observada neste estudo está em consonância com evidências amplamente documentadas na literatura científica. Estudo que observou a mortalidade por COVID-19 também destacou que indivíduos negros apresentaram no Brasil e em todas as regiões, exceto na Norte, maiores chances de mortalidade hospitalar^(15)^. Essa disparidade não pode ser atribuída exclusivamente a fatores biológicos, mas sim às desigualdades estruturais que afetam desproporcionalmente as populações negras^(16)^. As populações afro-americanas/negras e hispânicas enfrentam uma carga desproporcional de infecções por SARS-CoV-2 e mortalidade relacionada à COVID-19, mas não superior às taxas de letalidade. Revisão de literatura cita que a falta de acesso aos cuidados de saúde e os fatores de exposição desta população sustentam as disparidades observadas mais do que a suscetibilidade devido a condições comórbidas^(17)^.

As comorbidades cardiovasculares representaram 38% do total das comorbidades entre os sujeitos do estudo, com destaque para a hipertensão arterial sistêmica (34,2%). As comorbidades metabólicas representaram 27% do total de comorbidades, sendo Diabetes Mellitus a mais frequente (35%). No estudo de Zhou *et al*. (2020), as comorbidades estavam presentes em quase metade dos pacientes, sendo também a hipertensão a comorbidade mais comum, seguida de diabetes e doença coronariana. A probabilidade de morte hospitalar foi maior em pacientes com diabetes ou doença coronariana^(18)^.

No presente estudo, não se observou associação significativa entre óbito e as comorbidades. Uma possível explicação para esse achado pode estar relacionada ao tamanho da amostra ou à heterogeneidade das comorbidades. Além disso, é possível que fatores como resposta inflamatória individual, tempo de internação e acesso oportuno ao cuidado intensivo desempenhem um papel mais decisivo na mortalidade de jovens adultos, reduzindo o peso relativo das comorbidades isoladamente. Também deve ser considerado o viés de seleção, uma vez que todos os indivíduos da amostra já estavam em estado crítico no momento da admissão à UTI, o que pode ter homogeneizado o risco de mortalidade, independentemente do histórico clínico prévio.

Foi observada uma taxa de sepse de 64,7%, sendo a coinfecção bacteriana de foco pulmonar a mais frequente (57%). Taxa semelhante (60%) foi observada em estudo realizado em Nova Iorque, que envolveu 152 pacientes com COVID-19^(19)^. Outro estudo envolvendo pacientes com COVID-19 com idade média de 47 anos também mostrou a sepse de foco pulmonar como principal complicação entre os pacientes^(16)^. A pneumonia associada à ventilação mecânica (PAV) pode contribuir para tal taxa de infecção pulmonar. Estudo envolvendo pacientes com COVID-19 ventilados mecanicamente apresentou taxa de PAV de 54%^(20)^.

O uso de VM foi uma variável relacionada à mortalidade (p=0,001), como ratificam outros estudos, em que a taxa de mortalidade entre aqueles ventilados mecanicamente variou de 81% a 97%^(21-23)^.

Estudo realizado em Wuhan, que analisou a relação entre COVID-19 e VM no início do surgimento dos casos, destacou a VM como principal tratamento de suporte para pacientes críticos. Cerca de 71% necessitaram de VM, e 67% tiveram síndrome do desconforto respiratório agudo^(18)^.

A PEEP elevada, o tempo prolongado no uso de suporte respiratório causado pelo comprometimento das trocas gasosas e a hipoxemia grave do paciente com COVID-19 estão associado ao surgimento da LRA e ao aumento da morbimortalidade. Outra possível explicação para as taxas de LRA está diretamente relacionada ao uso de DVAs, que provocam vasoconstrição severa, atuando na diminuição do fluxo renal^(24)^.

Adultos jovens com COVID-19 que desenvolveram LRA, principalmente na estratificação KDIGO 3, apresentaram elevado número de óbito, assim como mostra literatura^(20)^. Estudo observou uma taxa de mortalidade entre aqueles que desenvolveram LRA bastante semelhante (44,63%)^(25)^.

A taxa de mortalidade entre aqueles que fizeram uso de TRS foi de 44%, e a literatura aponta para taxas que variam de 36% até 90%^(26)^.

A primeira atualização das diretrizes da campanha *Surviving Sepsis* sobre manejo de adultos com COVID-19 na UTI recomenda fortemente usar estratégia de pressão expiratória final positiva (PEEP) mais alta^(27)^.

Cerca de 65% dos adultos jovens fizeram uso de ao menos uma DVA. Estudos evidenciam que o uso de DVAs entre os pacientes com COVID-19 tratados na UTI varia de 28% a 94%^(28,29)^, sendo a noradrenalina o vasopressor de primeira linha, e a vasopressina, agente de segunda linha, assim como evidenciado no manejo clínico da amostra estudada^(30,31)^.

O achado de associação estatisticamente significativa entre o uso de heparina profilática (p=0,002) e corticosteroides (p=0,028), e o desfecho de óbito merece uma análise cuidadosa. Embora ambos os tratamentos estejam incluídos nas diretrizes terapêuticas da COVID-19 com o objetivo de reduzir complicações tromboembólicas e inflamatórias, a associação com maior mortalidade pode refletir viés de gravidade, ou seja, pacientes mais graves são mais propensos a receber essas intervenções. Uma combinação de resposta imune desregulada, com imunossupressão induzida por vírus e drogas, como os corticoides, pode contribuir para o surgimento das infecções secundárias no paciente com COVID-19, podendo culminar em maiores taxas de mortalidade^(32)^.

Entre os marcadores laboratoriais, a elevação dos valores de PCR (p=0,031), lactato (p=0,037) e linfócitos (p=0,046) esteve associado à mortalidade. No estudo de Zhou *et al*., os níveis de lactato se mostraram igualmente elevados em não sobreviventes, em comparação com sobreviventes, ao longo do curso clínico, assim como os níveis de D-dímero, diferentemente dos achados do presente estudo^(18)^.

Os níveis séricos de ácido lático elevados são comumente associados a mau prognóstico, sendo utilizado como um índice sensível de choque. Muitas condições podem aumentar sua concentração, incluindo metabolismo anaeróbico, glicólise, tempestade de citocinas e disfunção hepática. Dessa forma, um importante modo de detectar a sepse na COVID-19 é por meio de biomarcadores como o ácido lático^(22)^. Somado a isso, quando se observa a taxa de uso de DVAs obtida, pode-se inferir que muitos dos pacientes da amostra estudada evoluíram para choque séptico.

Em estudo realizado nos Estados Unidos que avaliou a ocorrência de sepse e hiperlactatemia em pacientes com COVID-19 internados em terapia intensiva, foi observado que o aumento do lactato não é um bom prognóstico na COVID-19, sendo que as taxas de mortalidade são mais altas do que as de outros tipos de sepse^(33)^.

Neste estudo, entre as variáveis estudadas, mostraram-se como fatores de risco para óbito na UTI o aumento dos níveis de lactato e os dias de uso de DVA.

Estudos apontaram para outros fatores de risco não evidenciados neste estudo, como sexo masculino, portador de hipertensão arterial sistêmica, diabetes, doença pulmonar obstrutiva crônica, doenças cardiovasculares ou oncológicas, altos níveis de PCR, e D-dímero. Ressalta-se que tais estudos não avaliaram a população adulta com estratificação por idade^(34,35)^.

Outros fatores podem ser decisivos para aumentar o risco de morte independentemente da idade. A ausência de políticas públicas de prevenção adequadas e a baixa capacidade de resolutividade da rede assistencial demonstram um contexto de grande desigualdade socioeconômica e iniquidade de acesso aos serviços de saúde, fatores que influenciam diretamente a mortalidade por COVID-19 no Brasil^(12)^.

### Limitações do estudo

As limitações do estudo incluem a perda de dados por ausência de registros nos prontuários eletrônicos. Além disso, os resultados podem não representar a realidade, uma vez que o estudo foi desenvolvido em centro único. Ademais, houve dificuldade para comparar achados a contento, pela escassez de estudos com resultados estratificados por faixa etária, envolvendo especificamente a população de adultos jovens ou pacientes no contexto exclusivo da UTI, reforçando a lacuna de conhecimento. O número de desfechos (n=29 óbitos) impõe restrições ao número de variáveis ajustadas simultaneamente, o que pode impactar a robustez do modelo.

### Contribuições para as áreas da enfermagem, saúde ou políticas públicas

Através deste estudo, foi possível traçar o perfil demográfico-clínico de adultos jovens internados em UTI por COVID-19, bem como conhecer variáveis associadas e preditoras de mortalidade nessa população. A análise de dados oriundos deste estudo se apresenta relevante, uma vez que acresce ao conhecimento sobre COVID-19 já existente, auxiliando no direcionamento de medidas gerenciais e assistenciais, e contribuindo para a prática clínica multiprofissional, com vistas a melhores desfechos.

## CONCLUSÕES

Obteve-se uma taxa de mortalidade de 27,6% entre os adultos jovens. As variáveis associadas ao óbito foram sexo (p=0,036), etnia (p=0,0057), procedência (p=0,045), instabilidade hemodinâmica (p=0,001), uso de DVA ( p=0,001), uso de VM (p=0,001), uso de heparina profilática (p=0,002) e uso de corticoide (p=0,028), além de aumento dos valores de PCR (p=0,031), lactato (p=0,037) e linfócitos (p=0,046).

As complicações associadas à mortalidade foram apresentar disfunção orgânica (p=0,009), sepse (p=0,009) e necessitar de TSR (p=0,001).

O aumento dos níveis de lactato e dos dias de uso de DVA se mostraram como fatores de risco para óbito na UTI.

## Data Availability

Os dados de pesquisa estão disponíveis no corpo do artigo.
